# Pseudoradial Nerve Palsy Caused by Acute Ischemic Stroke

**DOI:** 10.1177/2324709616658310

**Published:** 2016-07-13

**Authors:** Hassan Tahir, Vistasp Daruwalla, Jeremy Meisel, Samir E. Kodsi

**Affiliations:** 1 Temple University/Conemaugh Memorial Hospital, Johnstown, PA, USA; 2 Wyne State University/Detroit Medical Center, Detroit, MI, USA

**Keywords:** stroke, pseudoradial nerve palsy

## Abstract

Pseudoperipheral palsy has been used to characterize isolated monoparesis secondary to stroke. Isolated hand nerve palsy is a rare presentation for acute cerebral stroke. Our patient presented with clinical features of typical peripheral radial nerve palsy and a normal computed tomography scan of the head, which, without a detailed history and neurological examination, could have been easily misdiagnosed as a peripheral nerve lesion deferring further investigation for a stroke. We stress the importance of including cerebral infarction as a critical differential diagnosis in patients presenting with sensory-motor deficit in an isolated peripheral nerve pattern. A good history and physical exam can differentiate stroke from peripheral neuropathy as the cause of radial nerve palsy.

## Background

Isolated monoparesis has been used to describe selective hand motor deficit secondary to a cerebral stroke. The pseudoradicular sensory-motor defects are rare presentations of stroke and are frequently misdiagnosed as peripheral nerve lesions. Advances in brain imaging have improved our understanding of cortical brain mapping of hand.^1^ We report a case of left-sided pseudopalsy of the radial nerve presenting with numbness, tingling, and weakness of the extensors of the left hand in a 70-year-old male with history of hypertension and hemosiderosis. The definitive diagnosis was obtained by magnetic resonance imaging (MRI) of the brain, which revealed an embolic stroke in the right parieto-occipital lobe, involving the precentral gyrus along with moderate to severe right internal carotid artery stenosis.

## Case Presentation

A 70-year-old male with past medical history of hypertension only presented to the emergency department with left hand weakness and numbness for past 7 hours. He was unable to extend his wrist and fingers and had loss of sensation and paresthesia on the dorsum of the left hand. He had experienced a similar episode 3 days ago, which resolved in a few hours; the patient had initially attributed these symptoms to old age and muscle spasms. Today his symptoms did not resolve in the past few hours, hence he decided to come to the emergency room for further investigation. He denied any dizziness, lightheadedness, loss of consciousness, slurring of speech, ambulatory dysfunction, or difficulty maintaining balance. He denied any weakness or loss of sensation in any other extremity. His home medications were amlodipine, benazepril, and vitamin D_3_. He did have a family history significant for stroke and diabetes mellitus. He denied smoking or alcohol or drug abuse.

His vitals were temperature 36.9, heart rate 98, respiratory rate 20, blood pressure 140/65, oxygen saturation 98% on room, weight 68 kg, height 170 cm. On examination he was alert and oriented in time, place, and person. His gait and cranial nerves were intact. Motor examination demonstrated weakness of left wrist and finger extensor muscles with a muscle strength grading of −4. Strength in the left bicep, brachoradialis, tricep muscle, flexors of the fingers and wrist was normal. Sensory examination demonstrated mild decrease in sensation on the dorsal aspect of the left hand, predominantly in the anatomical snuff box. Strength and sensation in the other extremities were normal. Other systemic examinations were unremarkable.

## Investigations

Blood investigations revealed white blood cell count of 6.6, hemoglobin 15.9, hematocrit 45, platelet count 192, prothrombin time 10.8, international normalized ratio 1, partial thromboplastin time 26, creatinine 1, blood urea nitrogen 20, troponin 0.01, creatinine kinase total 106, chloride 112, sodium 136, potassium 4.1. Low-density lipoprotein was 140. Electrocardiogram demonstrated a sinus rhythm with nonspecific ST wave changes.

**Figure 1.**
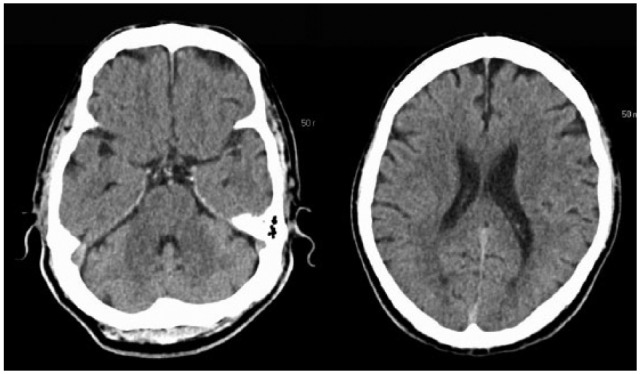
CT head done at the time of admission shows no ischemic stroke.

**Figure 2.**
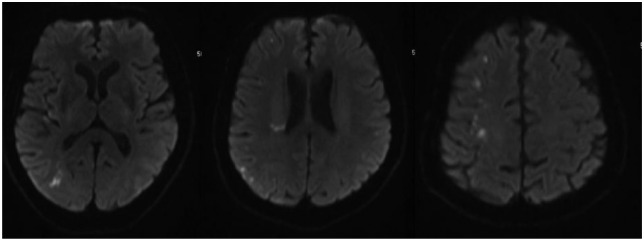
MRI brain without contrast shows small scattered foci of true restricted diffusion in the subcortical aspect of the right posterior parieto-occipital region, involving the precentral gyrus. Multiple other small infarcts are seen in the right frontoparietal convexity and right sided corona radiata adjacent to the posterior aspect of the right lateral ventricle.

Computed tomography (CT) scan of the head was normal (Figure1). MRI of the brain demonstrated small scattered foci of true restricted diffusion in the subcortical aspect of the right posterior parieto-occipital region, involving the precentral gyrus, which are suggestive of lacunar infarcts (Figure 2). Multiple other small infarcts were seen in the right frontoparietal convexity and right sided corona radiata adjacent to the posterior aspect of the right lateral ventricle (Figure 2). The findings are suggestive of multifocal stroke within the right MCA territory, and the likely etiology is atheroembolic from the ipsilateral ICA. No acute intracranial hemorrhage or midline shift was noted. The ventricles are normal in size with patent dural venous sinuses. The MRI brain gave an impression of multifocal small acute lacunar infarcts in the right cerebral hemisphere with true restricted diffusion, probably embolic in etiology.

**Figure 3.**
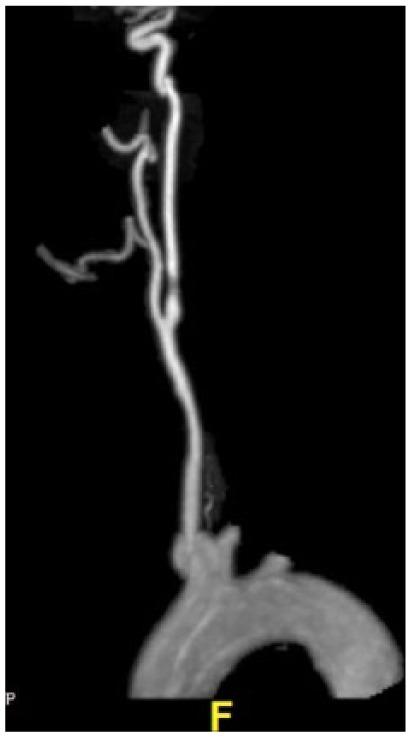
MRA of the neck showed 50% moderate stenosis by NASCET criteria within the proximal aspect of the right internal carotid artery approximately 1 cm from the right carotid bifurcation.

Magnetic resonance angiography (MRA) of the neck showed greater than 50%, moderate stenosis by NASCET criteria within the proximal aspect of the right internal carotid artery approximately 1 cm from the right carotid bifurcation (Figure 3).

**Figure 4.**
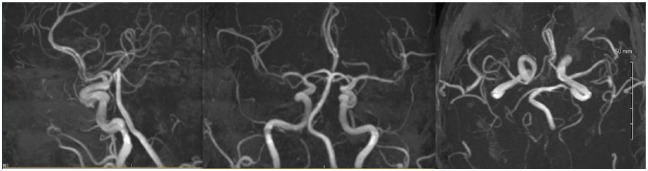
MRA of the head is normal.

MRA of the head was normal (Figure 4).

Transthoracic and transesophageal ultrasound (TES) of the heart were performed, which showed an ejection fraction of 55% but were negative for any clot. There was no patent foramen ovale on bubble study.

## Differential Diagnosis and Treatment

1. C7 radiculopathy and posterior cord brachial plexopathy
2. Radial or posterior interosseous neuropathy
3. Vasculitis leading to hyperacute mononeuropathies
4. Spinal cord disease like stenosis, trauma, or neoplasm
5. Hereditary neuropathy susceptible to pressure palsy

Our patient had presented after a time period of 3 hours; hence, thrombolytic therapy was not considered. After ruling out other causes of ischemic stroke, it was thought that the possible source of embolic stroke in our patient was carotid stenosis. Our patient underwent right carotid endarterectomy (CAE) for the greater than 50% occlusion seen on MRA of the neck, as per the American Academy of Neurology guidelines for CAE in symptomatic patients with greater than 50% carotid stenosis.^2^ Preprocedural aspirin was provided to the patient as recommended by the CAE guidelines in symptomatic patients.^2^ He was provided with a sling for his left hand along with appropriate physiotherapy and occupational therapy. He was started on atorvastatin for his elevated low-density lipoprotein of 140. Our patient was ambulating well and had no evidence of clot on TES; hence, prophylactic anticoagulation was not administered.

## Outcome and Follow-up

Subsequently our patient demonstrated improvement in his left hand weakness with the strength of the extensor of the wrist and hand graded as 4+ at discharge. At his 1-month follow-up our patient has some mild residual weakness in his left hand, which he believes to be improving.

## Discussion

Our patient presented with the classical features of a radial nerve palsy and was even diagnosed the same initially, but clinical history of recurring weakness lasting a few hours and meticulous neurological examination pointed toward an alternate diagnosis. The motor area of the hand has been mapped to a precentral knob in the precentral gyrus, which projects to the middle genu of the central sulcus. Cases of isolated motor lesion without sensory loss secondary to cerebral infarction have been reported. Lesions of the medial and lateral portions of the precentral knob have been related to the ulnar and radial side finger palsies, respectively.^3^

On literature review we found that Paciaroni et al had described lesions that were subcortical and belonged to the territory of posterior circulation among the 32 of their 51 patients (62.7%) with isolated monoparesis reviewed in about 6 years. Stroke-related isolated monoparesis were triggered by small artery disease in 40% of the patients. Hemodynamic infarct was suggested as a cause in 3 patients as boundary zones were affected.^4^ Carotid artery dissection leading to wrist drop has been reported recently following a borderzone infarct.^5^ Eight patients with cortical infarction in the region of precentral gyrus presented as isolated hand palsy in the study by Celebisoy et al.^6^ The bottom of the central sulcus, posterior bank of precentral gyrus, or precentral knob and upper portion of precentral gyrus have been suggested to be the motor hand area of the cerebral cortex location.^7^

Six patients with infarcts in the boundaries of anterior, middle, and posterior cerebral arteries along with severe carotid stenosis due to low basal blood flow in the parietal region was noted by Timsit et al. Babinski sign was absent but all presented with mild sensory symptoms and/or signs. Angular gyrus was affected in all except one patient.^8^

Our patient had presented beyond the set time period (3 hours) for intravenous thrombolytic therapy, and in many cases, the treatment is delayed secondary to misinterpretation of the nerve palsies as peripheral lesion. Hence, we stress the importance of maintaining a high index of clinical suspicion for acute cerebral infarct and carotid stenosis/dissection in patients with isolated nerve palsies without a history of pain to suggest vasculitis or waking from sleep with the deficit (compression) or fall (trauma), shoulder pain (neuralgic amyotrophy), or neck pain with radicular symptoms to offering timely and aggressive treatment.

## Learning Points/Take Home Messages

1. Isolated nerve palsies are frequently misdiagnosed as peripheral nerve lesions.
2. Acute cerebral infarction and carotid dissection should be considered as differential for isolated nerve palsies with recurrent history of transient weakness and resolution in someone with vascular risk factors like diabetes mellitus, hyperlipidemia, and hypertension.
3. Delay in the diagnosis of acute stroke or carotid dissection hinders adequate thrombolytic or invasive therapy necessary to prevent further progression of these lesions.
4. CT scan of the head may be normal in such cases.
5. MRI of brain with diffusion restricted and perfusion weighted imaging demonstrates mismatch regions, which have been suggested to denote ischemic brain tissue at risk. Advanced imaging techniques have improved accurate diagnosis of these lesions.
